# Preclinical evaluation of the CDK4/6 inhibitor palbociclib in combination with a PI3K or MEK inhibitor in colorectal cancer

**DOI:** 10.1080/15384047.2023.2223388

**Published:** 2023-06-16

**Authors:** Cha Len Lee, Mattia Cremona, Angela Farrelly, Julie A. Workman, Sean Kennedy, Razia Aslam, Aoife Carr, Stephen Madden, Brian O’Neill, Bryan T. Hennessy, Sinead Toomey

**Affiliations:** a Medical Oncology Group, Department of Molecular Medicine, Royal College of Surgeons in Ireland, Dublin, Ireland; b Data Science Centre, Royal College of Surgeons in Ireland, Dublin, Ireland; c Department of Radiation Oncology, St. Luke’s Radiation Oncology Centre, Beaumont Hospital, Dublin 9, Ireland

**Keywords:** Colorectal cancer, drug combinations, palbociclib, Gedatolisib, S6rp (S240/244)

## Abstract

**Background:**

Studies have demonstrated the efficacy of Palbociclib (CDK 4/6 inhibitor), Gedatolisib (PI3K/mTOR dual inhibitor) and PD0325901 (MEK1/2 inhibitor) in colorectal cancer (CRC), however single agent therapeutics are often limited by the development of resistance.

**Methods:**

We compared the anti-proliferative effects of the combination of Gedatolisib and Palbociclib and Gedatolisib and PD0325901 in five CRC cell lines with varying mutational background and tested their combinations on total and phosphoprotein levels of signaling pathway proteins.

**Results:**

The combination of Palbociclib and Gedatolisib was superior to the combination of Palbociclib and PD0325901. The combination of Palbociclib and Gedatolisib had synergistic anti-proliferative effects in all cell lines tested [CI range: 0.11–0.69] and resulted in the suppression of S6rp (S240/244), without AKT reactivation. The combination of Palbociclib and Gedatolisib increased BAX and Bcl−2 levels in *PIK3CA* mutated cell lines. The combination of Palbociclib and Gedatolisib caused MAPK/ERK reactivation, as seen by an increase in expression of total EGFR, regardless of the mutational status of the cells.

**Conclusion:**

This study shows that the combination of Palbociclib and Gedatolisib has synergistic anti-proliferative effects in both wild-type and mutated CRC cell lines. Separately, the phosphorylation of S6rp may be a promising biomarker of responsiveness to this combination.

## Introduction

Colorectal cancer (CRC) is the second most common cause of cancer death in Europe. It is estimated that by 2030 the global burden of CRC will increase by 60%, with 2.2 million new cases and one million deaths worldwide.^1^ In the metastatic CRC setting, compound agent chemotherapies given in combination with anti-EGFR (epidermal growth factor receptor) or anti-VEGF (vascular endothelial growth factor) monoclonal antibodies are standard clinical practice, providing improvement in patient outcomes reaching median overall survival (OS) between 29 and 36 months (in patients with *RAS* wild-type disease).^2,3^ However, more than 50% of patients eventually relapse and subsequent treatment options rarely offer high clinical impact, especially in patients with *RAS* mutations.^4^

Treatment for CRC is complex and often limited by resistance to therapy, which can be intrinsic or acquired. The crosstalk between the PI3K/AKT/mTOR (phosphatidylinositol−3-kinase/acutely transforming retrovirus/mammalian target of rapamycin) and MAPK/ERK (mitogen activated protein kinase) pathways is recognized as a key mechanism of resistance to oncology therapy.^5^ Other mechanisms leading to the development of resistance include 1) Formation of new secondary site resistance mutations within the target kinase; 2) Activation of escape bypass routes involving signaling pathways such as MAPK/ERK and PI3K/AKT/mTOR; 3) Dysregulation of downstream effectors; 4) Transformation into pro-metastatic phenotypes, which enable the cancer cells to survive the effects of treatment; 5) Immune adaption within the tumor microenvironment to enable cancer cell survival, via either immune-dependent or immune-independent processes.^6^ Theoretically, combined therapies can produce synergistic inhibition in a relatively safe manner to reduce multiple growth signal transmission responsible for the development of drug resistance, as compared to monotherapy. There is now a growing appreciation for using combination therapeutic approaches which can be exploited through multiple modalities such as radiotherapy, chemotherapy, immunotherapy, or targeted agents.

The PI3K/AKT/mTOR and MAPK/ERK signaling pathways are highly implicated in CRC pathogenesis with key mutations like *RAS, BRAF*, and *PIK3CA* arising from both pathways. There are numerous data from Phase I/II trials support the use of Palbociclib (CDK 4/6 inhibitor),^7–10^ Gedatolisib (PI3K/mTOR dual inhibitors),^11,12^ and PD0325901 (selective MEK1/2 inhibitor)^13–15^ as single agents in various types of cancers. Nonetheless, these inhibitors have limited cytoreductive effect when used as single agents because of drug resistance. As shown in breast cancer models, the combination of a CDK 4/6 inhibitor with a PI3K/mTOR inhibitor have produced synergistic treatment effects. This specific drug combination can overcome treatment-related resistance by preventing RSK activation and subsequent MAPK/ERK pathway activation.^7,16–18^ Currently, there are several active Phase I trials evaluating the combination of Palbociclib with Gedatolisib in patients with refractory malignancies including CRC. Similarly, another Phase Ib trial is evaluating the effectiveness of combining a different PI3K/mTOR dual inhibitor (Samotolisib) with a CDK4/6 inhibitor (Abemaciclib) in multiple common cancers.^19^ By extrapolating the available literature, we believe the approach of using Palbociclib with Gedatolisib to prevent the emergence of resistance in breast cancer is hypothetically applicable to other cancers including CRC. In parallel, there is strong preclinical evidence for the evaluation of co-inhibition using Palbociclib with PD0325901 in CRC.^13–15^ Of note, the combination of Gedatolisib with PD0325901 has previously been shown to have unacceptable toxicity in humans.^20^

In summary, treatment with a targeted therapy combination has multiple advantages over treatment with a single agent as anti-proliferative efficacy can be maximized within an acceptable overlapping drug toxicity limit. In comparison to monotherapy, combined drug therapies inhibit multiple-targets and have diverse cellular regulatory actions and are thus more likely to be effective in attenuating drug resistance pathways. This method has been exploited in various cancers, particularly in breast cancer. As we have noted, there is a research-gap in CRC, therefore there is a need to explore novel therapeutics using combinative drug approaches. We hypothesized that the combination of Palbociclib with either Gedatolisib or PD0325901 could produce synergistic benefits and have potential clinical relevance for the treatment of refractory CRC.

## Results

### Effects of Palbociclib, Gedatolisib, and PD0325901 in CRC cell lines

Drug concentrations used for the combination assays and the corresponding IC_50_ values of single-agent Palbociclib, Gedatolisib, and PD0325901 in CRC cell lines are summarized in Table 1. Arbitrarily defining a peak plasma concentration of 15 µM as a cut-off for sensitivity to Palbociclib, Caco−2, DLD−1, and LS1034 cell lines was resistant to Palbociclib (IC_50_ >15 µM). LS411N and SNUC4 cells were sensitive to Palbociclib (IC_50_: LS411N = 0.8 µM; SNUC4 = 1.7 µM). The LS1034 cell line was relatively resistant to Gedatolisib (IC_50_ = 7.2 µM). All other cell lines were more sensitive to Gedatolisib with IC_50_ values generally within nanomolar ranges (IC_50s_: LS411N = 76 nM; DLD−1 = 183 nM; SNUC4 = 400 nM; Caco−2 = 1200 nM). The cell lines also had high sensitivity to PD0325901 (IC_50s_: LS411N = 0.001 µM; LS1034 = 0.013 µM; SNUC4 = 0.014 µM; Caco−2 = 4.0 µM; DLD−1 = 13 µM). The Caco−2 cell line was relatively resistant to all drugs, in particular Palbociclib (IC_50_ >15 µM).

**Table 1.** The inhibitory concentration 50 (IC_50_) values and subsequent combination drug doses used for Palbociclib (CDK 4/6 inhibitor), Gedatolisib (PI3K/mTOR dual inhibitors), and PD0325901 (selective MEK 1/2 inhibitor) against the tested colorectal cancer cell lines with various mutational backgrounds. The IC_50_ values highlighted in bold are indicative of resistant cell lines to the drug. Each experiment was repeated 3–4 times. Cell line suppliers are ATCC=American Tissue Type Collection; KCLB=Korean Cell Line Bank.

Cell Line	Mutational Status	IC_50_ Palbociclib	IC_50_ PD0325901	IC_50_ Gedatolisib	Palbociclib + PD0325901 Doses	Palbociclib + Gedatolisib Doses
Caco−2 (ATCC HTB−37)	*Wild-Type*	**>15 µM**	4.0 µM	1200 nM	5 µM +5 µM	5 µM +1 µM
DLD−1 (ATCC CCL−221)	*KRAS* G13D *PIK3CA* E545K	**>15 µM**	13 µM	183 nM	2 µM +5 µM	2 µM +200 nM
LS411N (ATCC CRL−2159)	*BRAF* V600E	0.8 µM	0.001 µM	76 nM	2 µM +5 µM	2 µM +200 nM
LS1034 (ATCC CRL−2158)	*KRAS* A146T	**>15 µM**	0.013 µM	**7200 nM**	3 µM +500 nM	3 µM +400 nM
SNUC4 (KCLB 0000C4)	*PIK3CA* E545G	1.7 µM	0.014 µM	400 nM	3 µM +25 nM	3 µM +400 nM

### Effect of Palbociclib in combination with Gedatolisib or PD0325901 in CRC cells lines

**Figure 1.**
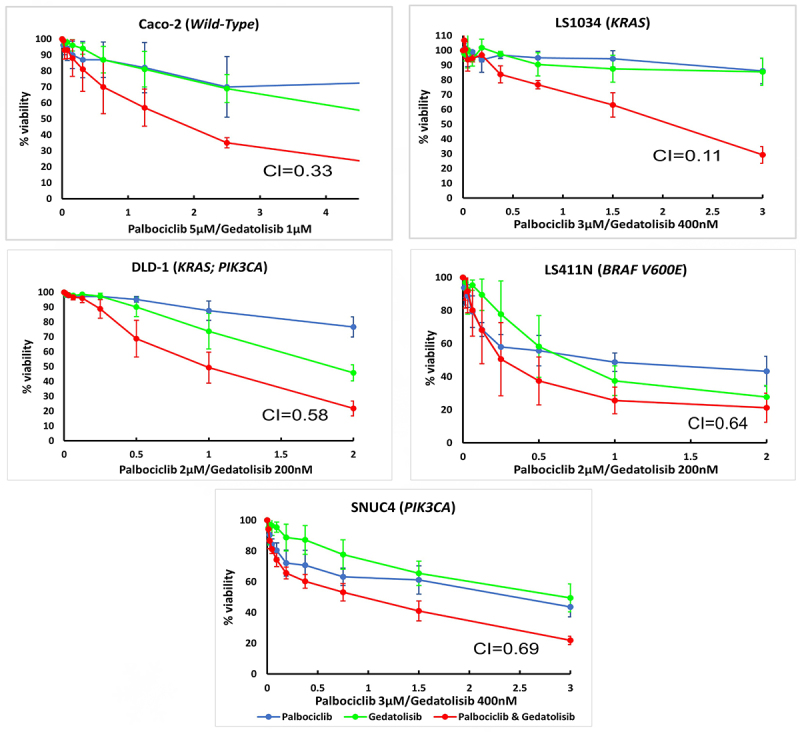
Cell growth inhibitory effects of Palbociclib, Gedatolisib and the combination of Palbociclib with Gedatolisib in (A) Caco −2, (B) LS1034, (C) DLD − 1, (D) LS411N and (E) SNUC4 cell lines. Each cell line was treated with increasing concentrations of Palbociclib, Gedatolisib and their combination at various fixed ratio doses which were pre-determined by the single agent IC_50_ values. The x-axis represents the combined drug doses in the ratio of Palbociclib’s dose. Cell viability was assessed using a 6-day acid phosphatase assay. The graphs show the mean cell growth ± standard error of mean (SEM) from a minimum of 3 repeats in each cell line. CI=Combination Index at effective dose 50.

Drug combination analysis showed that the combination of Palbociclib with Gedatolisib has a synergistic anti-proliferative effect in all CRC cell lines tested (Table 2; Figure 1). The combination of Palbociclib and Gedatolisib is highly synergistic in LS1034 cells (*KRAS* mutation; CI = 0.11). The combination of Palbociclib with Gedatolisib is also synergistic in DLD−1 (*KRAS* and *PIK3CA* mutated; CI = 0.58) and Caco−2 (*wild-type;* CI = 0.33) cells. The combination of Palbociclib with Gedatolisib is minimally synergistic in the LS411N (*BRAF* V600E mutated; CI = 0.64) and SNUC4 (*PIK3CA* mutated; CI = 0.69) cell lines.

**Table 2.** Combination indexes at effective dose 50 (CI) for two drug combinations, i.e., Palbociclib with Gedatolisib (P+G) and Palbociclib with PD0325901 (P+PD) tested in this study. The CI effects are in vitro drug response in five colorectal cancer cell lines. A CI < 0.9 is indicative of a synergistic effect, between 0.9 and 1.0 is additive, and > 1.1 is antagonistic.

Cell Line	Drug Combinations	CI
Caco−2	P+PD P+G	0.17 0.33
DLD−1	P+PD P+G	0.06 0.58
LS411N	P+PD P+G	14.7 *(antagonistic)* 0.64
LS1034	P+PD P+G	0.29 0.11
SNUC4	P+PD P+G	0.44 0.69

**Figure 2.**
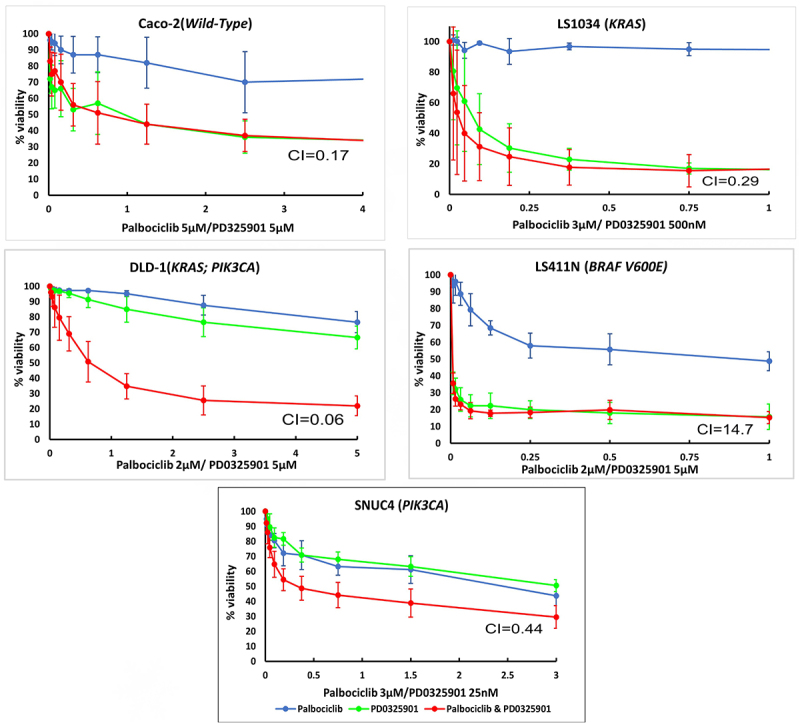
Cell growth inhibitory effects of the combination of Palbociclib, PD0325901 and the combination of Palbociclib with PD0325901 in (A) Caco −2, (B) LS1034, (C) DLD − 1, (D) LS411N, and (E) SNUC4 cell lines. Each cell line was treated with increasing concentrations of Palbociclib, PD0325901 and their combination at various ratio doses which were pre-determined by the single-agent IC_50_ values. The x-axis represents the combined drugs doses in the ratio of palbociclib’s dose. Cell viability was assessed using a 6-day acid phosphatase assay. The graphs showed the mean cell growth ± standard error of mean (SEM) values from minimum 3 repeats in each cell lines. CI=Combination Index at effective dose 50.

The combination Palbociclib with PD0325901 produces a synergistic growth inhibitory response in all cell lines, apart from LS411N cells (CI values: DLD−1 = 0.06; Caco−2 = 0.17; LS1034 = 0.29; SNUC4 = 0.44; LS411N = 14.7) (Table 2; Figure 2).

### Effect of the combination of Palbociclib with Gedatolisib on inhibition of the PI3K/AKT/mTOR pathway

We conducted RPPA analysis using 40 primary antibodies representing multiple nodes of the PI3K/AKT/mTOR, MAPK/ERK and intracellular apoptotic signaling pathways, following 4-h treatment with Palbociclib, Gedatolisib, and their combination in the Caco−2, DLD−1, LS1034, and SNUC4 cell lines.

**Figure 3.**
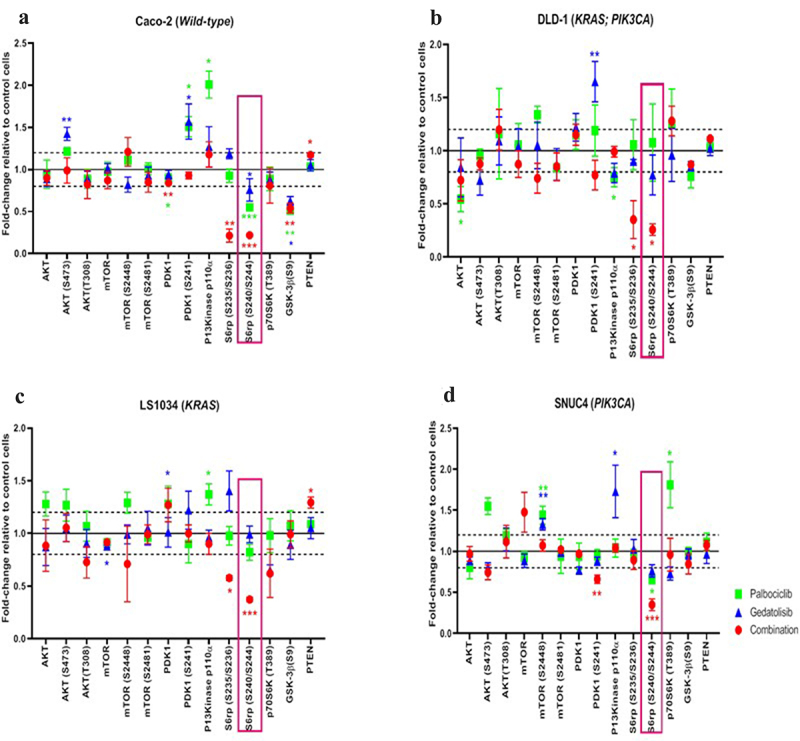
RPPA analysis displaying the levels of protein expression or phosphorylation of PI3K signaling pathway proteins in (A) Caco −2, (B) DLD − 1, (C) LS1034 and (D) SNUC4 cell lines following 4 hours treatment with Palbociclib, Gedatolisib and their combination. Fold changes shown have been normalized relative to the vehicle treated control cells. Error bars are representative of independent triplicate experiments. All p-values were generated by the Kruskal Wallis test. *p < .05, **p < .002, ***p < .001.

The combination Palbociclib with Gedatolisib showed synergistic inhibition of some components of the PI3K/AKT/mTOR pathway compared to other treatment arms (Figure 3). The combination of Palbociclib with Gedatolisib resulted in significant suppression of phosphorylated S6 Ribosomal protein S6rp (S240/244) across all cell lines [Caco−2 fold change= −4.55 ± 0.01, *p < .001*; DLD−1 fold change= −3.85 ± 0.05, *p = .012*; LS1034 fold change= −2.70 ± 0.02, *p < .001* and SNUC4 fold change= −2.86 ± 0.07, *p < .001*]. The combination Palbociclib with Gedatolisib also caused significant suppression of S6rp (S235/236) in Caco−2 [fold change= −4.70 ± 0.08, *p = .001*], DLD−1 [fold change= −2.85 ± 0.18, *p = .021*] and a close to significant suppression in LS1034 [fold change= −1.74 ± 0.02, *p* = .050], compared to vehicle control treated cells. The suppression of S6rp (S240/244) and S6rp (S235/236) was much more marked with the combination of Palbociclib with Gedatolisib than with either Palbociclib or Gedatolisib alone.

We did not observe increases in phosphorylated PDK1 (S241) or AKT (S473 and T308) with the combination Palbociclib with Gedatolisib, suggesting that there was no feedback activation of AKT signaling (Figure 3). In contrast, AKT (S473) and PDK1 (S241) levels did increase in the Caco−2 and DLD−1 cells following treatment with single-agent Gedatolisib, possibly reflective of feedback loop activity. In SNUC4 cells, PDK1 (S241) was significantly reduced by the combination Palbociclib with Gedatolisib [fold change= −1.52 ± 0.02, *p = .006*] compared to vehicle control treated cells. In comparison to vehicle control treated cells, the combination Palbociclib with Gedatolisib appeared to exert greater suppression of phosphorylated mTOR (S448) than Palbociclib or Gedatolisib alone, especially in DLD−1 cells [fold change= −1.18 ± 0.13, *p* = .236] and LS1034 cells [fold change= −1.41 ± 0.36,*p* = .316]. However, these changes were not statistically significant. There was no change in the levels of phosphorylated mTOR (S2481) in any of the cell lines treated with the combination Palbociclib with Gedatolisib compared to control. However, treatment with the combination Palbociclib with Gedatolisib significantly increased *PTEN* levels compared to control treated LS1034 cells [fold change = 1.29 ± 0.05, *p = .011*] and Caco−2 cells [fold change = 1.17 ± 0.02, *p = .022*].

### Effect of the combination of Palbociclib with Gedatolisib on apoptosis and cell cycle

**Figure 4.**
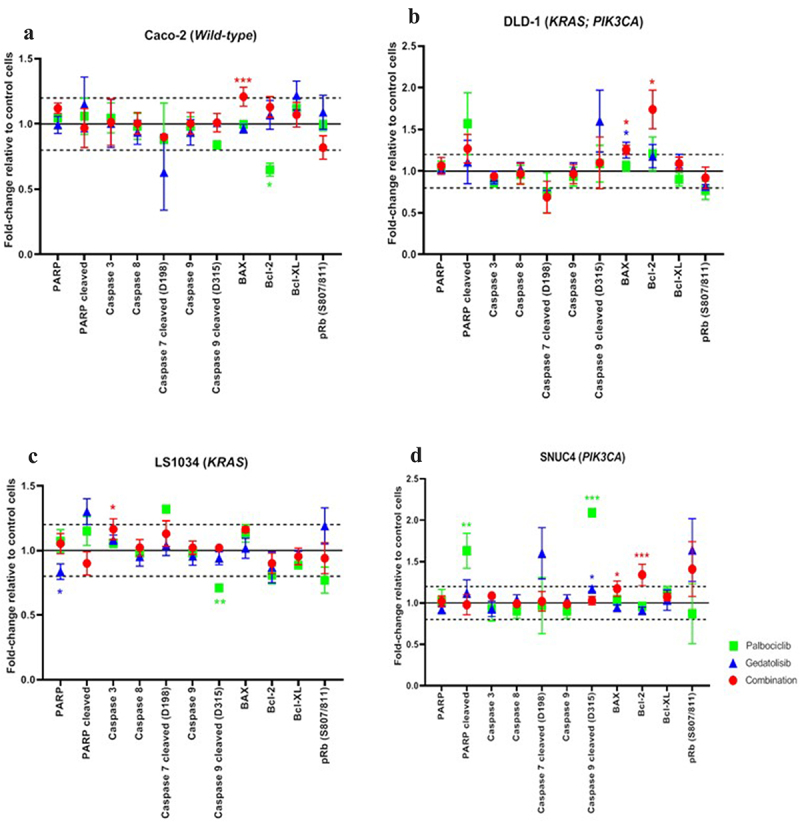
RPPA analysis displaying the levels of protein expression or phosphorylation of PI3K signaling pathway proteins in (A) Caco −2, (B) DLD − 1, (C) LS1034 and (D) SNUC4 cell lines following 4 hours treatment with Palbociclib, Gedatolisib and their combination. Error bars are representative of independent triplicate experiments. Fold changes shown have been normalized relative to the vehicle treated control cells. All p-values were generated by the Kruskal Wallis test. *p < .05, **p < .002, ***p < .001.

The expression and phosphorylation of the intracellular apoptotic signaling proteins were assessed to determine if the addition of Gedatolisib can enhance the actions of Palbociclib during cell cycle progression to increase apoptosis (Figure 4). In comparison to vehicle control treated cells and in contrast to single-agent Palbociclib or Gedatolisib, this resulted in increased levels of BAX and Bcl−2. The combination Palbociclib with Gedatolisib significantly increased BAX levels in DLD−1 [fold change = 1.26 ± 0.06, *p = .039*], SNUC4 [fold change = 1.17 ± 0.09, *p = .019*] and Caco−2 cells [fold change = 1.21 ± 0.07, *p < .001*]. We also observed significantly increased Bcl−2 levels in DLD−1 [fold change = 1.09 ± 0.08, p* = 0.017*] and SNUC4 [fold change = 1.07 ± 0.05, *p=<0.001*] cells treated with the drug combination, an effect that was not observed with single-agent Palbociclib or Gedatolisib.

The increase in BAX and Bcl−2 levels in DLD−1 and SNUC4 cells treated with the combination Gedatolisib with Palbociclib was not associated with an increase in caspase 3, caspase 8, cleaved caspase 7, cleaved caspase 9, and cleaved PARP levels, possibly indicating that the observed increases were not sufficient to induce total apoptosis. However, it is possible that the 4-h treatment timepoint used in our study was too early to measure the late proteomic alterations associated with apoptosis. Of interest, the addition of Gedatolisib to Palbociclib did not induce any changes in the phosphorylated Ribosomal protein (Rb (S807/811)).

### Effects of the combination of Palbociclib with Gedatolisib (P+G) on EGFR and the MAPK/ERK pathway

**Figure 5.**
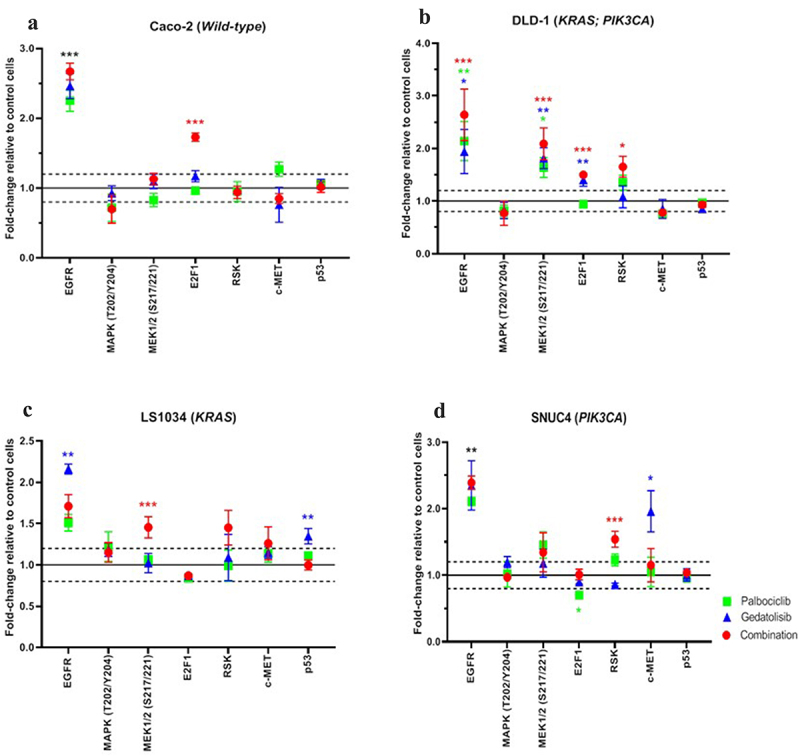
RPPA analysis displaying the levels of protein expression or phosphorylation of EGFR and MAPK signaling pathway proteins in (A) Caco −2, (B) DLD − 1, (C) LS1034 and (D) SNUC4 cell lines following 4 hours treatment with Palbociclib, Gedatolisib and their combination. Error bars are representative of independent triplicate experiments. Fold changes shown have been normalized relative to the vehicle treated control cells. All p-values were generated by the Kruskal Wallis test. *p < .05, **p < .002, ***p < .001.

One of the mechanisms for the development of resistance to PI3K-targeted inhibitors is the reactivation of membrane receptor tyrosine kinases (RTKs) and/or the MAPK/ERK signaling cascade. Our RPPA data showed an increase in the total EGFR in all cell lines after 4 h of treatment (Figure 5). Following treatment with the combination Palbociclib with Gedatolisib, EGFR levels were upregulated in all cell lines compared to vehicle control as follows: Caco−2 fold change = 2.67 ± 0.12; *p < .001*, DLD−1 fold change = 2.64 ± 0.49; *p < .001*, SNUC4 fold change = 2.39 ± 0.10; *p = .001* and LS1034 fold change = 1.71 ± 0.14; *p* = .072. Although MAPK(T202/Y204) phosphorylation did not increase following any treatment, MEK1/2(S217/221) phosphorylation did increase after single agent and combination treatments in DLD−1 cells, as did levels of E2F1, suggesting global activation of MAPK/ERK signaling, including with the combination treatment. The fold changes for MEK1/2(S217/221) were as follows: Combination Palbociclib with Gedatolisib = 2.09 ± 0.30; *p < .001*, Gedatolisib = 1.81 ± 0.20; *p = .003* and Palbociclib = 1.64 ± 0.19; *p = .013* whilst the fold changes for E2F1 were combination Palbociclib with Gedatolisib = 1.50 ± 0.03; *p < .001*, Gedatolisib = 1.40 ± 0.12; *p = .005* and Palbociclib = 0.94 ± 0.07; *p* = .845.

Of note, there was also an increase in Ribosomal S6 Kinase, in particular with the combination treatment compared to control arm, e.g.,, in DLD−1 [fold change = 1.65 ± 0.20; *p = .025*] and SNUC4 [fold change = 1.54 ± 0.12, *p < .001*] cells. This may also reflect MAPK/ERK reactivation, which occurs in some cell lines, particularly after treatment with Palbociclib and Gedatolisib in combination.

RPPA adjusted p-values after 4 h of treatment are shown in Supplementary Table S3. Additional RPPA analysis after 30 min of treatment was also carried out and p-values for the comparison of all antibodies measured between 30-min and 4-h treatment timepoints are shown in Supplementary Table S4.

## Discussion

Resistance to anti-cancer therapies evolves dynamically within 6–12 months of starting treatment due to various mechanisms, and combined drug treatment is a promising strategy for tackling resistance.^5,7–9,11,21,22^ In this study, we investigated the impact of combining Palbociclib (CDK 4/6 inhibitor), with either Gedatolisib (PI3K/mTOR dual inhibitor) or PD0325901 (selective MEK 1/2 inhibitor) on CRC cell line growth. We also investigated the proteomic effects of the combination Palbociclib with Gedatolisib in CRC cell lines with various mutational backgrounds to identify potential biomarker(s) for this novel therapy.

The IC_50_ values for single-agent Gedatolisib were between 76 and 7200 nM. These values were comparatively higher than previous studies, which reported IC_50_ values of less than 100 nM,^23,24^ although Caco−2, LS411N, LS1034, and SNUC4 were not included in this analysis. We also observed a higher range of IC_50_ values for single-agent Palbociclib in our cell lines, in comparison to other studies.^25^ PD0325901 demonstrated a relatively low IC_50_ range in our cell lines, suggesting more innate sensitivity to MEK inhibition.

Our results demonstrated that the combination Palbociclib with either Gedatolisib or PD0325901 exhibits synergistic anti-proliferative effects, relative to the single agents, in all cell lines including LS1034 (*KRAS* mutated) and DLD−1 (co-occurring *KRAS* with *PIK3CA* mutated) cells. These are important findings since *KRAS* and *PIK3CA* mutations occur in approximately 60% and 20% of CRC, respectively.^26^ In LS411N (*BRAF* V600E mutated) cells, the combination Palbociclib with Gedatolisib demonstrated mild synergy whilst the combination Palbociclib with PD0325901 failed to show any synergistic effects (CI = 0.64 versus 14.7). In LS1034 cells, we noted a synergistic effect with both drug combinations, however the combination Palbociclib with Gedatolisib appears to be superior (CI = 0.11 versus 0.29). In the Caco−2 *wild-type* cells, both drug combinations demonstrated comparable synergistic effects. Taken together, we considered the combination of Palbociclib with Gedatolisib to be of higher priority for future clinical development, compared to the combination of Palbociclib with PD0325901 and thus decided to focus our proteomic investigations on Palbociclib with Gedatolisib. The subnanomolar IC_50_ range of Gedatolisib makes it a more favorable companion drug than PD0325901 to avoid excessive overlapping toxicities. Furthermore, strong efficacy and safety data is already available from Phase I clinical trials involving the combination Palbociclib with Gedatolisib.^7,8^ In these trials, Palbociclib (125 mg) was administered orally, daily for 3 weeks with Gedatolisib (110 mg) administered intravenously once during the 4-week cycle.

In our RPPA study following 4 h of exposure to the combination Palbociclib with Gedatolisib, we observed suppression of S6rp (S240/S244) across all cell lines. There was also a significant suppression of S6rp (S235/S236) in Caco−2, DLD−1, and LS1034 cells. This suppression was much more marked with the combination Palbociclib with Gedatolisib than with either single agent alone. Suppression of S6rp was not associated with any increased expression or phosphorylation of AKT, indicating that there was no upstream PI3K reactivation in response to the combination. We thus believe that the combination Palbociclib with Gedatolisib acts primarily at the level of mTOR, which is a downstream effector of the PI3K/AKT signaling cascade. Our results also show that the combination Palbociclib with Gedatolisib likely exhibits stronger inhibition of the PI3K/AKT/mTOR pathway in comparison to control or single-agent therapy. For example, we observed increased levels of AKT (S473) and PDK1 (S241) in some cell lines with single-agent treatment, possibly reflective of feedback loop activity.

In view of the global suppression of S6rp (S240/S244) in all tested cell lines, this may be a promising predictive marker of clinical responsiveness for this combination therapy. As reported by Iwenofu *et al.*, ^27^ S6rp is considered a better surrogate biomarker of mTOR activity in comparison to p70S6K, also known as Ribosomal protein S6 kinase beta−1 (S6K1). This is because p70S6K has structural similarity to p90S6K, which is not phosphorylated by mTOR.^27,28^ As a key component of the PI3K/AKT signaling cascade, mTOR plays a crucial role in the regulation of energy metabolism and protein synthesis by directly activating p70S6K.^29–32^ p70S6K is a serine-threonine kinase that controls S6rp phosphorylation at five serine residues (S235, S236, S240, S244, and S247), leading to initiation of protein synthesis.^33^ In contrast to the S240/244 residues which are solely regulated by p70S6K, phosphorylation at the S235/S236 residues is controlled by multiple kinases including p70S6K, p90RSK, and PKA.^34^ This may explain the suppression of S6rp (S235/S236) which was significant in Caco−2, DLD−1, and LS1034 but not in SNUC4 cells, in contrast to S6rp (S240/S244).

Emerging experimental data has suggested utilizing PI3K/mTOR inhibitors to induce non-cell autonomous actions by modulating signal transduction during G_1_ to S phases, leading to increased cell death.^35–38^ Interestingly, we did not observe any increase in pRb (S807/S811) following treatment with the combination Palbociclib with Gedatolisib. This is consistent with results previously described by Vora *et al*. ^39^ It appears that PI3K inhibition suppress AKT phosphorylation but can fail to suppress CDK 4/6 activity, as measured by Rb phosphorylation.^40^ Nonetheless, the regulation of Rb function by phosphorylation during cell cycle is not fully understood. Rb in mammalian cells has 15 known phosphorylation sites and it appears that Rb phosphorylation at specific sites is required for Rb to regulate apoptosis.^41,42^ *In vitro* studies have shown that phosphorylation of Rb at S608/S795 in addition to S807/S811 may play a role in the induction of apoptosis.^43^ Furthermore, there is evidence to suggest that dephosphorylation of Rb has been widely observed during apoptosis.^44^ This may explain the equivocal level of pRb (S807/S811) we observed in our RPPA analysis.

Unlike the single agents, the combination Palbociclib with Gedatolisib induced (early) pro-apoptotic effects, as demonstrated by increased BAX and Bcl−2 levels in most cell lines tested. The increase in both markers was significant in DLD−1 and SNUC4 cells but was not significant in LS1034 cells, suggesting that the magnitude of effect may be dependent on the cells’ mutational status. We did not observe any increase in caspase 3, caspase 8, cleaved caspase 7, cleaved caspase 9, or cleaved PARP levels, which are indicative of total apoptosis. However, it is possible that the 4-h timepoint used for our analysis was too early to evaluate the proteomic alterations related to the late stage of apoptosis.

Finally, we observed EGFR and RSK upregulation in all cell lines after 4 h of drug treatment, which may be associated with upstream MAPK/ERK reactivation. Total EGFR and RSK upregulation were observed with both mono- and combination therapy in some of the cell lines. This suggests that the mechanism promoting resistance to PI3K-targeted inhibitors (which was Gedatolisib in this study) include feedback loops, which lead to reactivation of membrane RTKs and the contralateral MAPK/ERK pathway. This further supports our hypothesis that a multiple target inhibition strategy, rather than single agent, is a better therapeutic option to prevent the development of resistance. The combined drug inhibition using Palbociclib with Getatolisib would simultaneously target the upstream and downstream effectors including the interconnection points between the PI3K/AKT/mTOR and MAPK/ERK pathways. It provides broader inhibition on the vast interconnection of both pathways, while minimizing feedback loops activation.

It is important to note the limitations of our study. First, it was an *in vitro* study and limited to five cell lines. Second, not all specific exon mutations were tested in this study, specifically *PIK3CA* mutations in exon 20 which may be biologically more relevant than exon 9 mutations from an epidemiology standpoint.^45–47^ Third, the RPPA analysis with 40 preselected antibodies was performed at only two timepoints (i.e., . 30 minutes and 4 h) post-drug exposure. The RPPA results are dependent on the selected timepoints, and it is likely we will be able to capture additional proteomic information if longer timepoints are used. The specific mechanism of synergism for the combination Palbociclib with Gedatolisib could not be completely defined in this study; however, from our RPPA analysis there are several possible mechanisms, including more complete inhibition of protein synthesis-related signaling (e.g., S6rp(S240/S244)) and increased activation of early apoptotic signaling. Despite these limitations, our study has produced evidence to support further *in vivo* evaluation, which is in progress.

In summary, the novel combination Palbociclib with Gedatolisib displays clear synergistic anti-proliferative effects in both *wild-type* and mutated CRC cell lines, relative to the single agents. Our results offer good rationale for further *in vivo* study and clinical development of Palbociclib and Gedatolisib as emerging therapeutics in metastatic CRC patients. S6rp (S240/S244) may be a marker of responsiveness for this novel combination therapy.

## Methods

### Cell lines

Five human CRC epithelial cell lines with commonly found mutational variations were used in this study (Table 1). Caco−2 (wild type for *PIK3CA*, *KRAS*, and *BRAF* mutations), DLD−1 (*KRAS/PIK3CA* mutated), LS411N (*BRAF* mutated), and LS1034 (*KRAS* mutated) were obtained from the American Tissue Type Collection (ATCC, USA). SNUC4 cells (*PIK3CA* mutated) were obtained from the Korean Cell Line Bank (KCLB, South Korea). The mutational status of the cell lines was determined using the Cancer Cell Line Encyclopedia (CCLE), and mutations were verified by us using the MassARRAY system (Agena Bioscience).

Caco−2 cells were wild type for mutations in PI3K/AKT/mTOR and MAPK/ERK pathways. DLD−1 cells have *KRAS* G13D and *PIK3CA* E545K mutations. LS411N cells have a *BRAF* V600E mutation. LS1034 cells have a *KRAS* A146T mutation. SNUC4 cells have a *PIK3CA* E545G mutation. The cell line identity was confirmed by DNA fingerprinting (Biosciences). Cell lines were mycoplasma tested before and after *in vitro* experiments. Species information, source and culture details are shown for all cell lines in Supplementary Table S1.

### Drug inhibitors

Palbociclib (CDK 4/6 inhibitor), Gedatolisib (PI3K/mTOR dual inhibitors), and PD0325901 (selective MEK 1/2 inhibitor) were purchased directly from Selleckhem (Houston, TX, USA). All drugs were prepared in 100% dimethylsulfoxide (DMSO) at stock concentrations of 10 mM, 5 mM, and 10 mM, respectively. The two drug combinations tested were Palbociclib with Gedatolisib versus Palbociclib with PD0325901.

### Proliferation assays and drug combination analysis

The acid phosphatase assay was used to test the anti-proliferative effects of Palbociclib, Gedatolisib and PD0325901, alone and in combination in each cell line. This was performed over 6 days period, as previously described.^48^ Cells were plated at 1 × 10^4^ cells/mL into 96-well plates (100 µL per well) and incubated at 37°C with 5% CO_2_ for 24 h. Two hundred microliters of sterile H_2_O was added around the edges of the plate to prevent it from drying out. Following 24-h incubation, drugs were added at the indicated concentrations and incubated at 37°C for 5 days (120 h). On Day 6, all the drug was removed, washed, and processed for absorbance measurement at 405 nm using a 96-well plate reader. Inhibition of proliferation was calculated relatively to untreated controls to obtain the dose of half maximal inhibitory concentration (IC50). A minimum number of triplicate biological assays was performed for each experiment. IC50 values were calculated using CacuSyn software. The individual inhibitor IC50 values were used for dosing guidance in the subsequent drug combination analysis. The same cell number (1×10^4^ cells/mL) was used for the drug combination analysis. Drug concentrations used for the combination assays are shown in Table 1.

### Protein extraction and Reverse Phase Protein Array (RPPA)

4 × 10^5^ cells were seeded in 6-well plates and grown until confluent. The total protein was extracted using 100 µl lysis buffer (15% NACL 1 M, 1% Triton-X 100, 5% TRIS, 14% phosphatase inhibitors 7×, 65% dH_2_O), as previously described by us.^49,50^ Protein was quantified by the bicinchoninic acid (BCA) assay and stored at −80◦C until analysis. The combination Palbociclib with Gedatolisib was selected for RPPA investigation because this combination demonstrated synergism in all cell lines tested in our study.

Cell lysates with a protein concentration of 1.5 µg/µL for each replicate were prepared. Before RPPA processing, each sample was solubilized in sodium dodecyl sulfate (SDS) sample buffer (40% Glycerol, 8% SDS, 0.25 M Tris-HCL, pH 6.8, 50 nM Bond-breaker TCEP solution) and heated to 80°C for 5 min. RPPA analysis was carried out using triplicate biological replicates following 4-h treatment with Palbociclib, Gedatolisib, and their combination in Caco−2, DLD−1, LS1034, and SNUC4 cell lines. The cell lines were treated at the same fixed ratio doses used in the drug combination proliferative analysis. Details of all primary antibodies used for RPPA analysis are detailed in Supplementary Table S2.

### Statistical analysis

Microsoft Excel software was used to record all the raw datasets. CalcuSyn software version 3.1 (Biosoft) was used to calculate IC_50_ and combination index at effective dose 50 (CI) values. The CI values were determined using the Chou-Talalay equation on CalcuSyn. A CI < 0.9 is considered synergistic, between 0.9 and 1.0 is additive and > 1.1 is antagonistic. Each experiment was repeated 3–4 times. For the RPPA experiments, the mean and standard error of mean (SEM) were calculated from three biologically independent protein samples analyzed on the same RPPA slide. The mean and SEM were normalized to the vehicle-treated control samples. Based on our internal precision studies, this allows us to detect changes in protein expression with a coefficient of variance (CV) of less than 20%.^51^ To evaluate the effect of the combination treatment, a one-way analysis of variance (ANOVA) with Tukey’s multiple-comparison test was used (GraphPad PRISM version 8). To compare the effects of Palbociclib, Gedatolisib, and their combination treatment on protein expression and phosphorylation, the Kruskal–Wallis non-parametric test was used. *P* values of < 0.05 were considered statistically significant.

## Supplementary Material

Supplemental Material

## Data Availability

The original contributions presented in the study are included in the article/Supplementary Material. Further data are available from the corresponding author upon request.
