# Supplementary material for: Preclinical evaluation of the CDK4/6 inhibitor palbociclib in combination with a PI3K or MEK inhibitor in colorectal cancer
Source: Cancer Biol Ther. 2023 Jun 16;24(1):2223388. doi: 10.1080/15384047.2023.2223388 (PMC10281467; doi:10.1080/15384047.2023.2223388)
Supplement: Supplemental Material [file KCBT_A_2223388_SM1473.docx]

**Supplementary table 1:** Full information of the colorectal cancer epithelial cell lines used in this study. The cell lines mutational information was determined using Cancer Cell Line Encyclopaedia (CCLE) and mutations were verified using the Agena MassARRAY platform. All cell lines were maintained at 37°C with 5% CO_2_. Cell lines were Mycoplasma tested before and after the *in vitro* experiments.

| Cell Line | Mutational Status | Media | Tissue Type | Doubling time |
| --- | --- | --- | --- | --- |
| Caco-2  (ATCC HTB-37) | *Wild-Type* | EMEM  +20% FBS  +1% P/S | Colon,  Colorectal adenocarcinoma | 62 hours |
| DLD-1  (ATCC CCL-221) | *KRAS* G13D  *PIK3CA* E545K | RPMI 1640 +10%FBS  + 1% P/S | Colon, Dukes type C, colorectal adenocarcinoma | 24 hours |
| LS411N  (ATCC CRL-2159) | *BRAF* V600E | RPMI 1640 + 10% FBS  + 1% P/S | Caecum, Dukes type B, colorectal carcinoma | 24 hours |
| LS1034  (ATCC CRL-2158) | *KRAS* A146T | RPMI 1640 + 10% FBS  +1% P/S | Caecum, Dukes type C, colorectal carcinoma | 24 to 33 hours |
| SNUC4  (KCLB 0000C4) | *PIK3CA* E545G | RPMI 1640 + 10% FBS  + 1% P/S | Colon,  Colorectal adenocarcinoma | 24 hours |

RPMI 1640=Rowell Park Memorial Institute 1640 medium (Sigma-Aldrich, USA, Cat.# R8758); FBS=fetal bovine serum (Gibco, USA Cat.# 10270-106); EMEM=Eagle’s Minimum Essential Medium (ATCC, USA Cat.# 30-2003); P/S=Penicillin/Streptomycin (Gibco, USA, Cat.#15070-063). ATCC=American Tissue Type Collection; KCLB=Korean Cell Line Bank

**Supplementary table 2:** List of primary antibodies used in the RPPA experiments.


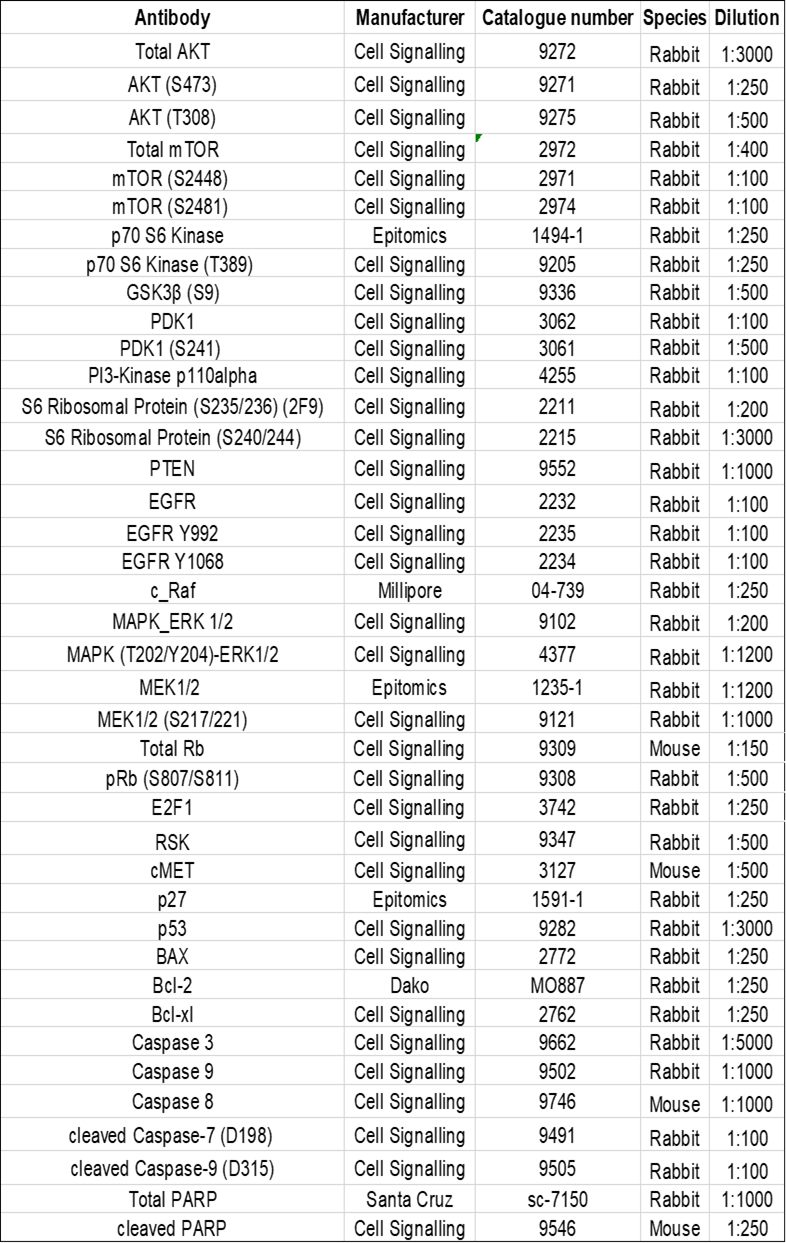


**Supplementary table 3:** Adjusted p-values following multiple testing for the antibodies used for Reverse Phase Protein Array (RPPA) analysis following 4 hours of treatment with Palbociclib (P), Gedatolisib (G) and the combination (P+G) in comparison to DMSO-treated control cells. DMSO= dimethylsulfoxide.

|  | Caco-2 | | | DLD-1 | | | LS1034 | | | SNUC4 | | |
| --- | --- | --- | --- | --- | --- | --- | --- | --- | --- | --- | --- | --- |
|  | P | G | P+G | P | G | P+G | P | G | P+G | P | G | P+G |
| AKT | 0.941 | 0.852 | 0.865 | **0.013** | 0.392 | 0.101 | >0.999 | 0.982 | 0.976 | 0.280 | 0.670 | 0.080 |
| AKT (S473) | 0.065 | **0.002** | 0.998 | 0.972 | 0.196 | 0.766 | 0.135 | 0.979 | 0.959 | 0.233 | 0.895 | 0.363 |
| AKT (T308) | 0.323 | 0.361 | 0.066 | 0.942 | 0.986 | 0.689 | 0.996 | 0.873 | 0.264 | 0.438 | 0.665 | 0.984 |
| mTOR | 0.931 | 0.992 | 0.110 | 0.968 | 0.994 | 0.278 | 0.100 | **0.019** | 0.105 | 0.971 | 0.867 | 0.415 |
| mTOR (S2448) | 0.757 | 0.365 | 0.255 | 0.097 | 0.779 | 0.236 | 0.325 | >0.999 | 0.316 | **0.002** | **0.010** | 0.996 |
| mTOR (S2481) | 0.999 | 0.886 | 0.542 | 0.279 | 0.402 | 0.368 | 0.987 | 0.948 | 0.999 | 0.984 | >0.999 | **<0.001** |
| P70S6K (T389) | 0.956 | 0.950 | 0.849 | 0.775 | >0.999 | 0.753 | 0.995 | 0.277 | 0.255 | **0.010** | 0.340 | 0.918 |
| GSK3β (S9) | **0.005** | **0.021** | **0.006** | 0.429 | 0.786 | 0.844 | 0.984 | 0.921 | >0.999 | >0.999 | 0.987 | 0.919 |
| PDK1 | **0.048** | 0.346 | **0.007** | 0.284 | 0.084 | 0.284 | 0.970 | **0.037** | 0.961 | 0.930 | 0.381 | **0.019** |
| PDK1 (S241) | **0.017** | **0.010** | 0.937 | 0.365 | **0.001** | 0.224 | 0.868 | 0.610 | >0.999 | 0.775 | 0.322 | **<0.001** |
| PI3K p110α | **0.046** | 0.875 | 0.947 | 0.036 | 0.076 | 0.997 | **0.032** | 0.986 | 0.800 | 0.997 | 0.035 | 0.971 |
| S6rp (S235/236) | 0.931 | 0.539 | **0.011** | 0.998 | 0.927 | **0.021** | 0.992 | 0.090 | 0.050 | 0.930 | 0.993 | 0.904 |
| S6rp (S240/244) | **<0.001** | **0.012** | **<0.001** | 0.245 | 0.878 | **0.012** | 0.090 | 0.995 | **<0.001** | **0.025** | 0.124 | 0.807 |
| PTEN | 0.881 | 0.695 | **0.022** | 0.939 | 0.985 | 0.259 | 0.583 | 0.497 | **0.011** | 0.894 | 0.986 | 0.997 |
| EGFR | **<0.001** | **<0.001** | **<0.001** | **0.002** | **0.010** | **<0.001** | 0.241 | **0.001** | 0.072 | **0.006** | **0.002** | >0.999 |
| c_RAF | 0.510 | 0.692 | 0.057 | 0.379 | 0.253 | 0.993 | 0.220 | 0.998 | 0.929 | 0.746 | 0.972 | 0.939 |
| MAPK-ERK1/2 | 0.438 | 0.829 | 0.462 | 0.637 | 0.902 | 0.343 | 0.998 | 0.404 | >0.999 | 0.627 | 0.976 | 0.988 |
| MAPK (T202/Y204) | 0.084 | 0.845 | 0.055 | 0.610 | 0.465 | 0.365 | 0.167 | 0.382 | 0.427 | >0.999 | 0.119 | 0.996 |
| MEK 1/2 | 0.061 | 0.497 | 0.452 | >0.999 | 0.583 | 0.558 | 0.797 | 0.362 | 0.990 | 0.296 | 0.070 | 0.992 |
| MEK 1/2 (S217/221) | 0.244 | 0.592 | 0.355 | **0.019** | **0.004** | **<0.001** | 0.924 | 0.675 | **<0.001** | 0.155 | 0.841 | 0.775 |
| Rb | **<0.001** | **<0.001** | **0.034** | 0.254 | 0.087 | 0.497 | **<0.001** | **<0.001** | **0.001** | 0.641 | **0.020** | 0.604 |
| pRB (S807/811) | >0.999 | 0.877 | 0.487 | 0.322 | 0.529 | 0.902 | 0.100 | 0.241 | 0.822 | 0.938 | 0.112 | 0.052 |
| E2F1 | 0.872 | 0.236 | **<0.001** | 0.845 | **0.005** | **<0.001** | 0.192 | 0.388 | 0.316 | **0.027** | 0.656 | 0.832 |
| RSK | 0.836 | 0.963 | 0.819 | 0.281 | 0.951 | **0.025** | 0.591 | 0.655 | 0.097 | 0.088 | 0.382 | 0.999 |
| cMET | 0.221 | 0.344 | 0.700 | 0.314 | 0.727 | 0.407 | 0.861 | 0.838 | 0.540 | 0.995 | **0.011** | >0.999 |
| P53 | 0.876 | 0.955 | 0.999 | 0.744 | 0.095 | 0.605 | 0.543 | **0.010** | >0.999 | 0.925 | >0.999 | 0.647 |
| BAX | >0.999 | 0.916 | **<0.001** | 0.862 | **0.048** | **0.039** | 0.524 | 0.996 | 0.339 | 0.884 | 0.611 | 0.996 |
| Bcl-2 | **0.027** | 0.933 | 0.580 | 0.742 | 0.798 | **0.017** | >0.999 | 0.938 | 0.951 | 0.852 | 0.346 | **0.006** |
| Bcl-xl | 0.540 | 0.160 | 0.884 | 0.679 | 0.555 | 0.757 | 0.459 | 0.997 | 0.905 | 0.901 | >0.999 | 0.998 |
| Caspase 3 | 0.988 | 0.996 | >0.999 | 0.162 | 0.539 | 0.681 | 0.702 | 0.380 | **0.041** | 0.069 | 0.964 | 0.593 |
| Caspase 9 | 0.986 | 0.686 | >0.999 | 0.871 | 0.980 | 0.762 | 0.924 | 0.953 | 0.935 | 0.536 | 0.922 | **<0.001** |
| Caspase 8 | 0.986 | 0.686 | >0.999 | 0.819 | 0.982 | 0.799 | 0.924 | 0.953 | 0.935 | 0.376 | 0.965 | 0.950 |
| c_Caspase 7 (D198) | 0.962 | 0.359 | 0.966 | 0.570 | 0.123 | 0.064 | 0.206 | 0.997 | 0.817 | >0.999 | 0.092 | **0.001** |
| C_Caspase 9 (D315) | 0.070 | 0.998 | 0.811 | 0.054 | 0.983 | 0.983 | **0.002** | 0.675 | 0.973 | **<0.001** | **0.028** | 0.323 |
| PARP | 0.731 | 0.999 | 0.107 | 0.881 | 0.969 | 0.709 | 0.452 | **0.028** | 0.669 | 0.932 | 0.514 | 0.459 |
| C_PARP | 0.954 | 0.778 | 0.993 | 0.187 | 0.998 | 0.692 | 0.212 | 0.077 | 0.938 | **0.007** | 0.808 | 0.968 |

**Supplementary Table 4:** The comparison of the mean fold-change of the antibodies measured at 30-minute and 4-hour treatment timepoints in RPPA analysis. Each treatment arm was compared with the same treatment arms of the different timepoint. The p-values were calculated with the two-way ANOVA test. The p<0.05 is considered statistically significant.

| **Antibodies** | **Caco-2** | **DLD-1** | **LS1034** | **SNUC4** |
| --- | --- | --- | --- | --- |
| Total AKT | 0.5641 | 0.4618 | 0.5641 | 0.6348 |
| AKT (S473) | 0.2836 | 0.3633 | 0.0985 | 0.2991 |
| AKT (T308) | 0.5034 | 0.2023 | 0.3413 | 0.3559 |
| Total mTOR | 0.4688 | 0.7971 | 0.1646 | 0.6938 |
| mTOR (S2448) | >0.9999 | 0.1034 | 0.3196 | 0.1930 |
| mTOR (S2481) | 0.5508 | 0.3257 | 0.8083 | **0.0255** |
| S6rb (S235/S236) | 0.7397 | 0.6127 | 0.5766 | 0.8625 |
| S6rb(S240/S244) | 0.5414 | 0.1378 | 0.6865 | 0.4545 |
| P70S6K(T389) | 0.5912 | 0.3611 | 0.4545 | 0.4599 |
| GSK3β(S9) | 0.8295 | 0.1886 | 0.5999 | 0.9027 |
| PDK1 | >0.9999 | 0.5440 | **0.0427** | 0.7257 |
| PDK1(S241) | 0.1778 | 0.7074 | 0.6332 | 0.6673 |
| PI3Kinase p110α | 0.7565 | 0.2811 | 0.9029 | 0.6027 |
| PTEN | 0.7949 | 0.3738 | 0.5540 | 0.0558 |
| MAPK_ERK1 | 0.8643 | 0.3228 | 0.5079 | 0.3438 |
| MAPK(T202/Y204) | 0.0866 | 0.1907 | 0.7863 | 0.1690 |
| EGFR | 0.0667 | **0.0229** | 0.0777 | 0.0652 |
| Total MEK1/2 | 0.3201 | 0.2987 | 0.4831 | 0.5935 |
| MEK1/2 (S217/221) | **0.0349** | 0.6703 | 0.4612 | 0.6754 |
| c_RAF | 0.2747 | 0.3936 | 0.4931 | 0.8197 |
| E2F1 | 0.3824 | 0.3359 | 0.1927 | 0.2636 |
| Total Rb | 0.5144 | 0.4733 | 0.4105 | 0.8681 |
| pRb (S807/811) | 0.2761 | 0.2256 | 0.5467 | 0.7172 |
| RSK | 0.8214 | 0.4837 | 0.2896 | 0.9910 |
| BAX | 0.2386 | 0.2327 | 0.4154 | 0.4739 |
| Bcl-2 | 0.4173 | 0.1829 | 0.1603 | 0.9067 |
| Bcl-xl | 0.3267 | 0.6329 | 0.3875 | 0.4352 |
| Caspase 3 | 0.8912 | 0.3528 | 0.5139 | 0.7568 |
| Caspase 9 | 0.1286 | **0.0306** | 0.3261 | 0.8721 |
| Caspase 8 | 0.3691 | 0.1039 | 0.3873 | 0.9776 |
| c_Caspase 7 (D198) | 0.1362 | 0.1749 | 0.4481 | 0.8623 |
| c_Caspase 9 (D315) | 0.1979 | 0.6391 | 0.1113 | 0.3182 |
| Total PARP | 0.6321 | 0.4334 | 0.9962 | 0.0870 |
| c_PARP | 0.4813 | 0.9620 | 0.9674 | 0.1393 |
| p53 | 0.4359 | 0.1411 | 0.6944 | 0.2414 |
| cMET | 0.2233 | 0.0846 | 0.2556 | 0.8252 |
